# Factors Associated With the Presence of Foveal Bulge in Eyes With Resolved Diabetic Macular Edema

**DOI:** 10.3389/fmed.2021.755609

**Published:** 2022-01-07

**Authors:** Qiaowei Wu, Yijun Hu, Baoyi Liu, Zhanjie Lin, Yu Xiao, Xiaomin Zeng, Ying Fang, Ying Yan, Ya Ye, Ming Yan, Zhen Huang, Honghua Yu, Yanping Song, Siwen Zang

**Affiliations:** ^1^The First School of Clinical Medicine, Southern Medical University, Guangzhou, China; ^2^General Hospital of Central Theater Command, Wuhan, China; ^3^Aier Institute of Refractive Surgery, Refractive Surgery Center, Guangzhou Aier Eye Hospital, Guangzhou, China; ^4^Aier School of Ophthalmology, Central South University, Changsha, China; ^5^Department of Ophthalmology, Guangdong Provincial People's Hospital, Guangdong Academy of Medical Sciences, Guangzhou, China

**Keywords:** complication of diabetic retinopathy, diabetic macular edema (DME), foveal bulge, optical coherence tomography (OCT), central foveal thickness (CFT), visual acuity (VA)

## Abstract

**Purpose:** To evaluate factors associated with the presence of foveal bulge (FB) in resolved diabetic macular edema (DME) eyes.

**Methods:** A total of 165 eyes with complete integrity of ellipsoid zone (EZ) at the fovea and resolved DME were divided into two groups according to the presence of FB at 6 months after intravitreal injection of ranibizumab treatment. Best-corrected visual acuity (BCVA), central foveal thickness (CFT), outer nuclear layer (ONL) thickness, height of serous retinal detachment (SRD) and non-SRD, and inner segment (IS) and outer segment (OS) lengths of the two groups were measured and compared at baseline and each follow-up. The correlations between the presence of FB and pre- and post-treatment factors were determined by logistic regression analysis.

**Results:** At baseline, BCVA was significantly better, and CFT and incidence and height of SRD were significantly lower in the FB (+) group (all *P* < 0.05). At 6 months, FB was present in 65 (39.39%) eyes. Post-treatment BCVA was significantly better and OS length was significantly longer in the FB (+) group at 6 months (all *P* < 0.05). Multivariate analysis identified younger age, better BCVA, and lower CFT before treatment as significant predictors of the existence of FB at 6 months (all *P* < 0.05). At 6 months, better BCVA and longer OS length were significantly correlated with the existence of FB (all *P* < 0.05).

**Conclusions:** Factors associated with the presence of FB after the resolution of DME include younger age, better baseline BCVA and lower baseline CFT, and better post-treatment BCVA and longer post-treatment OS length.

## Introduction

Diabetic macular edema (DME), affecting 1.4–12.8% of diabetic patients globally, is one of the primary causes of vision impairment in diabetic patients (1–3). Although different effective treatments are available for DME, the resolution of DME is not always followed by satisfactory visual recovery, and there may be paradoxical responses (4). Several studies have shown that only 18–45% of DME patients with good anatomical responses gained more than 15 letters after 2 years of treatment (5–8). Therefore, it is important to explore anatomical biomarkers of visual recovery in patients with DME. Various optical coherence tomography (OCT) biomarkers, such as disruption in the integrity of ellipsoid zone (EZ) and external limiting membrane (ELM), were proved significantly correlated with visual outcomes of patients with DME (9). However, the visual prognosis is still unsatisfactory in some patients with resolved DME and complete integrity of EZ at the fovea. The same issue also occurred in patients with macular edema secondary to branch retinal vein occlusion (BRVO-ME), DME with serous retinal detachment (SRD-DME), and rhegmatogenous retinal detachment (10–12). In these patients, foveal bulge (FB) can serve as a biomarker of better visual recovery after retinal reattachment or complete edema resolution (10–12). Therefore, we propose that FB has an important impact on the visual recovery after the resolution of DME.

Diabetic macular edema could be classified into 3 types according to the morphologic characteristics on OCT examination, such as SRD, diffused retinal thickening (DRT), and cystoid macular edema (CME) types (9, 13, 14). The pathogenesis of each DME type may differ from each other. For example, SRD mainly involves the disruption of outer blood-retinal barriers (BRB), whereas DRT and CME are caused by the breakdown of inner BRB (9, 15, 16). Therefore, these DME types can be further classified into SRD and non-SRD (i.e., DRT and CME) categories according to their different pathogenesis (17). Our previous study has demonstrated the association between the restoration of FB and severity of SRD in eyes with resolved SRD-DME (12), but the effect of non-SRD on the restoration of FB is still unknown.

Previous studies have shown that the photoreceptor in the fovea could be affected by severe macular edema, resulting in photoreceptor dysfunction and loss of photoreceptor cells (11). Moreover, the photoreceptor damage resulting from severe DME can lead to the absence of FB (11). Another previous study about the variation in FB with age has suggested that FB was more likely to be observed in younger healthy individuals and the height of FB decreased with age (18). Thus, we propose that the existence of FB may be associated with various factors, such as age and severity of DME, in eyes with resolved DME. This study aimed to evaluate factors related to the presence of FB after the resolution of DME.

## Materials and Methods

### Subjects

This retrospective study included 165 eyes (114 patients) with resolved DME in the Department of Ophthalmology at Guangdong Provincial People's Hospital between January 2017 and May 2020. Initially, each eye received best-corrected visual acuity (BCVA) measurement with a decimal chart and clinical examination, such as spectral-domain OCT (SD-OCT) scanning (Spectralis; Heidelberg Engineering, Heidelberg, Germany), intraocular pressure (IOP) measurement, and slit-lamp biomicroscope. At 1, 3, and 6 months after the loading treatment, all included eyes underwent BCVA measurement and SD-OCT scanning. The study was approved by the Institutional Review Board of Guangdong Provincial People's Hospital and was conducted according to the Declaration of Helsinki. Since the study is about the retrospective analysis of outcomes of a standard DME treatment and no individual patient could be identified from the data, formal informed consent was waived.

Eyes with DME affecting the central fovea at baseline and resolved DME with complete integrity of EZ at 6 months were included. We included eyes that were untreated or received previous anti-vascular endothelial growth factor (VEGF) or pan-retinal photocoagulation (PRP) at least 6 months ago and eyes with the central foveal thickness (CFT) over 275 μm and BCVA between 0.3 and 1.0 logarithm of the minimal angle of resolution (LogMAR) (20/200–20/40) at baseline (12, 19). Eyes with macular edema secondary to other causes, such as retinal artery/vein occlusion, age-related macular degeneration, and polypoidal choroidal vasculopathy, eyes with macular ischemia, glaucoma or IOP > 21 mmHg, a history of macular grid photocoagulation or vitrectomy, refractive error over 6 diopters (D), severe cataracts, or previously treated with PRP or intravitreal or periocular injection <6 months were excluded (12, 17). We also excluded eyes with unsatisfactory SD-OCT images resulting from poor patient cooperation or media opacity.

### Treatment

All included eyes received a loading dose of 3 monthly consecutive 0.5-mg intravitreal injections of ranibizumab (IVR) treatment. After the loading treatment, patients were followed on monthly basis and received 1 IVR injection if they met any of the following criteria: (A) CFT increases by ≥ 100 μm; (B) BCVA decreases by ≥ 0.1 LogMAR; or (C) the decrease of BCVA attributed to newly formed SRD or intraretinal cyst, based on the surgeons; or (D) the decrease of BCVA due to enlargement of previous SRD or intraretinal cyst, based on the surgeons (9, 12, 17). IVR injection was suspended if either of the following criteria was met: (A) BCVA ≤ 0.0 LogMAR (20/20) observed at the two last consecutive follow-ups; or (B) stable BCVA over three consecutive follow-ups that include the current follow-up evaluation, specifically no improvement of BCVA due to IVR injections at the two last consecutive follow-ups (9, 12, 17).

### Measurement and Classification of DME on OCT Images

**Figure 1:**
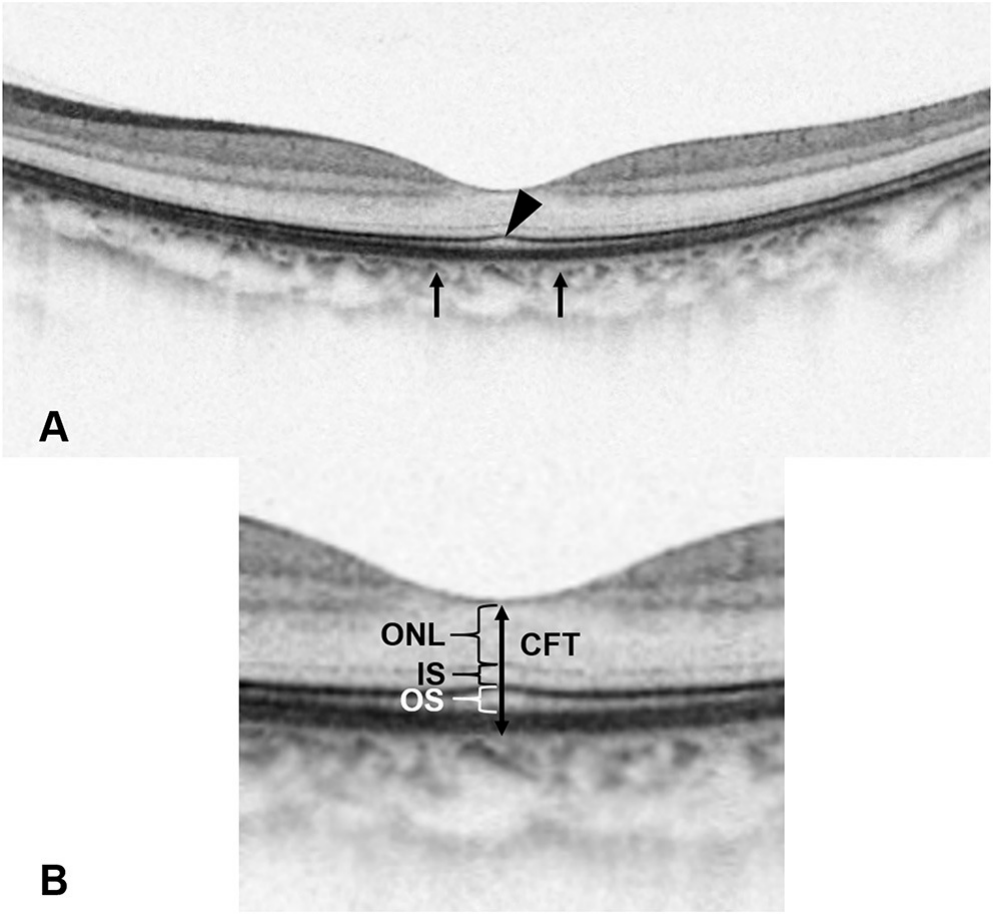
The definition of foveal bulge (FB) and optical coherence tomography (OCT) measurements. **(A)** A FB (arrowhead) located at the intact ellipsoid zone line is shown on the OCT image. FB is defined as a dome-shaped structure of the ellipsoid zone (EZ) where the length of the outer segment (OS) located at the central fovea is ≥10 μm longer than the mean OS length located at 250 μm nasal and temporal from the central fovea (arrows). **(B)** Enlarged view. Central foveal thickness is measured as the vertical distance between the outer border of the retinal pigment epithelium (RPE) and the surface of the internal limiting membrane (ILM) at the central fovea. Outer nuclear layer thickness is measured as the vertical distance between the outer border of the external limiting membrane (ELM) and the outer border of ILM. Inner segment length is measured as the vertical distance between the outer border of EZ and the outer border of ELM. OS length is measured as the vertical distance between the inner border of RPE and the outer border of EZ.

**Figure 2:**
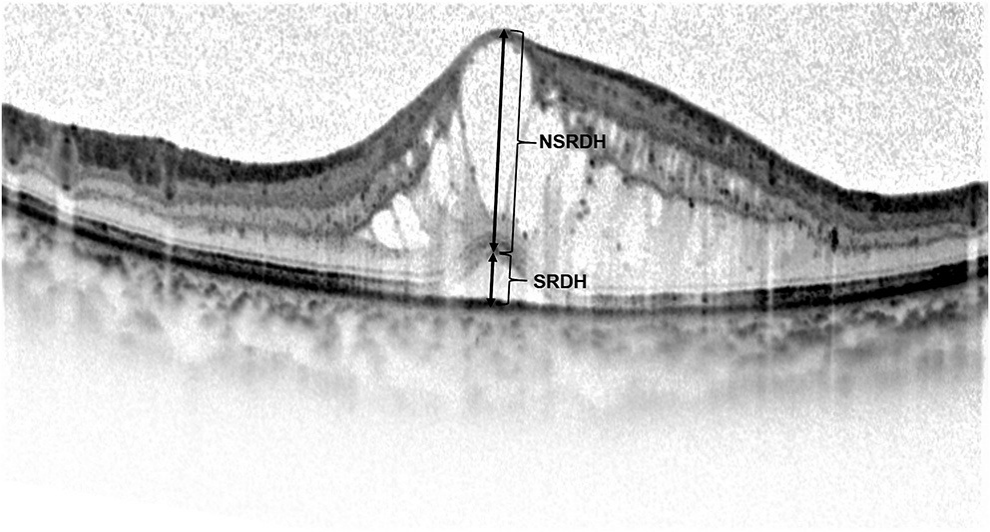
Manual measurement of the height of serous retinal detachment (SRDH) and non-serous retinal detachment (NSRDH) located at the central fovea on the optical coherence tomography image. The SRDH is measured as the vertical distance at the fovea between the signal from the anterior boundary of the retinal pigment epithelium-choriocapillaris region and the top of subretinal fluid. The NSRDH is measured as the vertical distance at the fovea between the top of subretinal fluid and the surface of the internal limiting membrane.

A set of high-speed SD-OCT scans (Spectralis; Heidelberg Engineering, Heidelberg, Germany) was obtained using a custom 20° × 20° volume acquisition protocol. With this protocol, we can obtain 25 horizontal and central vertical cross-sectional B-scan images, each composed of 512 A-scans (12, 17). According to the morphologic characteristics on OCT examination, DME was classified into three types (i.e., SRD, DRT, and CME). SRD type was defined as an optically clear space between the retinal pigment epithelium (RPE) and retina and a shallow elevation of the retina (9, 12, 14). DRT type was defined as sponge-like retinal swelling of the macula with reduced intraretinal reflectivity (9, 12, 14). CME type was defined as highly reflective septa separating cystoid-like cavities and low reflective intraretinal cystoid spaces in the macular area (9, 12, 14). Three DME types were further classified into two categories: SRD and non-SRD (i.e., DRT and CME) according to their different pathogenesis (17). Two ophthalmologists (QW and BL) exported the horizontal SD-OCT images through the fovea of all the included eyes for independent reading and measurement of CFT, outer nuclear layer (ONL) thickness, height of SRD (SRDH) and non-SRD (NSRDH), and inner segment (IS) and outer segment (OS) length (Figures 1, 2) (12). If there were discordance between the two ophthalmologists, arbitration was performed by a retinal specialist (HY) to generate the final decision.

**Figure 3:**
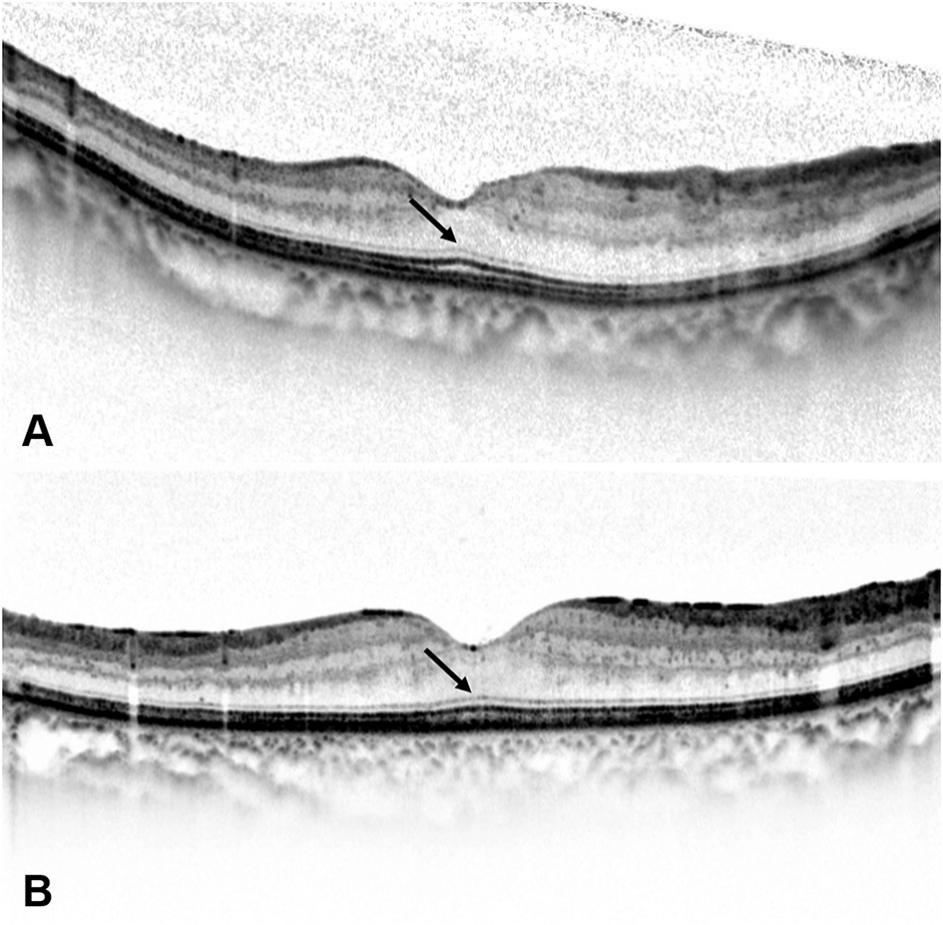
At 6 months after the loading treatment, **(A)** the spectral-domain optical coherence tomography (SD-OCT) image of a 49-year-old woman with a BCVA of 0.0 LogMAR (20/20) shows a resolution of diabetic macular edema (DME), a long outer segment (OS) length, and the existence of foveal bulge (FB) (arrow). **(B)** The SD-OCT image of a 43-year-old woman with a BCVA of 0.1 LogMAR (20/25) also shows a resolution of DME, a long OS length, and the existence of FB (arrow).

**Figure 4:**
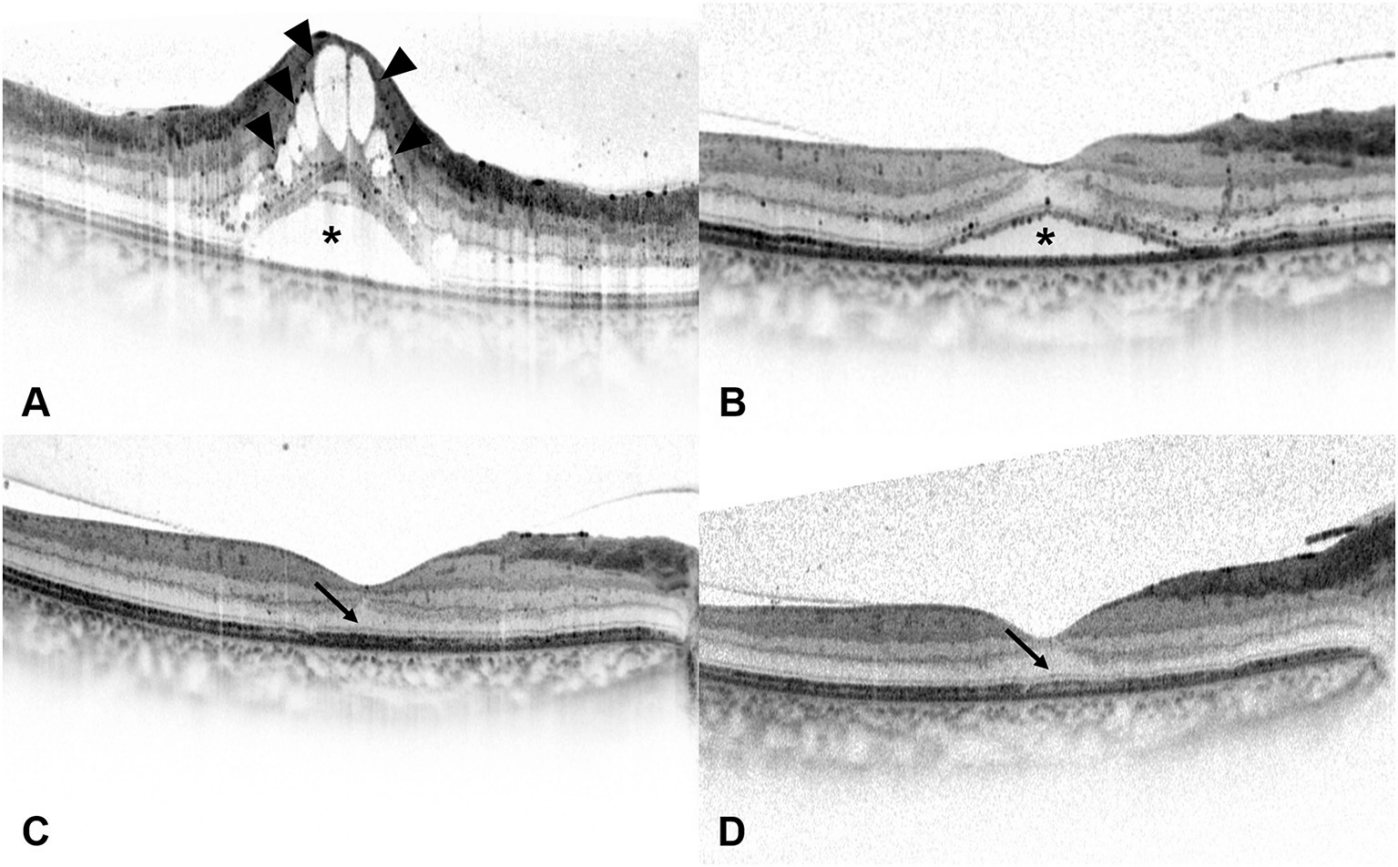
Spectral-domain optical coherence tomography (SD-OCT) images of a 54-year-old man with diabetic macular edema (DME). **(A)** Best-corrected visual acuity (BCVA) of this patient was 0.7 LogMAR (20/100) at baseline and the SD-OCT image showed the presence of serous retinal detachment (*) and cystoid macular edema (arrowheads). Disruption of ellipsoid zone (EZ) and external limiting membrane (ELM) could be seen at the central fovea. **(B)** At 1 month after the loading treatment, BCVA of this patient improved to 0.52 LogMAR (20/63), and the SD-OCT image showed residual subretinal fluid (*) and the absorption of intraretinal cystoid fluid. **(C)** At 3 months after the loading treatment, BCVA of this patient improved to 0.3 LogMAR (20/40), and the SD-OCT image showed the absorption of subretinal fluid and the presence of intact ELM and EZ within the central fovea. Foveal bulge (FB) could not be detected (arrow). **(D)** At 6 months after the loading treatment, central foveal thickness further decreased on the SD-OCT image, and BCVA of this patient improved to 0.22 LogMAR (20/32). FB still could not be detected (arrow), while the continuity of EZ could be seen at the central fovea.

Based on the existence of FB at 6 months after the loading treatment, the included eyes were divided into the FB (+) group and the FB (–) group. As a qualitative OCT parameter, FB was defined as a dome-shaped structure of the EZ where the length of OS located at the central fovea is ≥10 μm longer than the mean OS length located at 250 μm nasal and temporal from the central fovea (Figure 1) (10, 12). SD-OCT images of typical cases of the two groups are shown in Figures 3, 4.

The two ophthalmologists manually measured CFT, ONL thickness, SRDH, NSRDH, IS length, and OS length at the central fovea in a masked manner (Figures 1, 2). Mean values of OCT measurements of the two ophthalmologists were used for statistical analysis. CFT was measured as the vertical distance between the outer border of RPE and the surface of the internal limiting membrane (ILM) (10, 12, 17). SRDH was measured as the vertical distance at the fovea between the anterior boundary of the RPE-choriocapillaris region and the top of subretinal fluid, while NSRDH was measured as the vertical distance at the fovea between the top of subretinal fluid and the surface of ILM (12, 17). ONL thickness was measured as the vertical distance between the outer border of ELM and the outer border of ILM (10, 12). IS length was measured as the vertical distance between the outer border of EZ and the outer border of ELM (10, 12). OS length was measured as the vertical distance between the inner border of RPE and the outer border of EZ (10, 12).

### Statistical Analysis

Best-corrected visual acuity with a decimal chart was converted to the LogMAR for statistical analyses. All values are presented as mean ± SD. We used the SPSS 20.0 (SPSS. Inc, Chicago, IL, USA) to perform all statistical analyses and calculated intra-class coefficient (ICC) values to assess the reproducibility of OCT measurements between the two ophthalmologists (QW and BL). The mean values and frequency of parameters between the two groups were compared using the unpaired Mann-Whitney test and the chi-square Test, respectively, after confirming the data normality.

Univariate and multinomial logistic regression analyses were performed to identify possible baseline predictors for the presence of FB and to evaluate the correlations between the presence of the FB and post-treatment factors. In the univariate analysis, the association between the detection of the FB and each variable was solely examined. Subsequently, stepwise backward multivariate regression analysis was performed with the following parameters: age, baseline BCVA, baseline CFT, SRDH, detection of SRD and disrupted ELM at baseline, post-treatment BCVA, and post-treatment OS length. The variables significantly associated with the detection of the FB in the univariate analysis or showing statistically significant differences between the two groups in the unpaired Mann-Whitney test and the chi-square Test were introduced as independent variables. The multinomial logistic regression analysis generates an odds ratio (OR) for each category of the dependent variable relative to the reference category. The OR value includes the 95% CI allowing estimating the degree of accuracy. All continuous variables were categorized as the quartile-categorical variables for univariate and multinomial logistic regression analyses. For all the tests and analyses, a value of *P* < 0.05 was considered statistically significant.

## Results

### Baseline Demographic Characteristics

This study included 165 eyes with complete integrity of EZ at the central fovea and completely resolved DME. At 6 months, FB was present in 65 (39.39%) eyes. In the FB (+) group, there were 47 eyes (72.31%) received previous anti-VEGF or PRP at least 6 months ago, compared to 79 eyes (79.00%) in the FB (–) group (*P* = 0.323). There were no significant differences in other baseline characteristics between the FB (+) group and the FB (–) group (Table 1). Moreover, the proportion of eyes from the FB (+) group was not significantly different between eyes with and without prior treatment (37.30% vs. 46.15%, *P* = 0.323). The interobserver ICC for the measurement of ONL thickness was 0.914, for IS length was 0.855, and for OS length was 0.872, indicating that the OCT measurements between the two ophthalmologists (QW and BL) with good reproducibility.

**Table 1:** Baseline demographic characteristics.

	**FB (+) (*n* = 65)**	**FB (–) (*n* = 100)**	***P***
Mean age (SD), years	58.00 (9.52)	60.28 (10.80)	0.065^*^
Male, n (%)	36 (55.38)	48 (48.00)	0.354^†^
Mean intraocular pressure (SD), mmHg	14.43 (2.72)	14.89 (2.83)	0.249^*^
Mean time since diagnosis of diabetes (SD), years	8.94 (5.36)	7.91 (5.19)	0.173^*^
Mean time since first diagnosis of DME (SD), months	8.72 (4.56)	8.18 (5.19)	0.196^*^
Mean HbA1c (SD), %	8.16 (1.57)	8.12 (2.26)	0.277^*^
Diabetic retinopathy stage, n (%)			0.777^†^
Non-proliferative diabetic retinopathy	43 (66.15)	64 (64.00)	
Proliferative diabetic retinopathy	22 (33.85)	36 (36.00)	
Previous treatment more than 6 months ago	47 (72.31)	79 (79.00)	0.323^†^
Photocoagulation treatment, n (%)	23 (35.38)	31 (31.00)	0.558^†^
Mean number of ranibizumab injections (SD), n	4.23 (1.52)	4.29 (1.35)	0.572^*^

*FB, foveal bulge; DME, diabetic macular edema; HbA1c, glycosylated hemoglobin; SD, standard deviation*.

*Statistically analysis:*

* *Unpaired Mann-Whitney Test;*

† *Chi-square Test*.

### Pre-Treatment BCVA and Optical Coherence Tomographic Parameters

At baseline, BCVA was significantly better, and CFT, SRDH, incidence of SRD, and disrupted ELM were significantly lower in the FB (+) group than the FB (–) group. NSRDH has no significant difference between the two groups (Table 2). BCVA of the FB (+) group was 0.38 ± 0.18 LogMAR, and BCVA of the FB (–) group was 0.59 ± 0.19 LogMAR (*P* < 0.001). CFT of the FB (+) group was 442.65 ± 155.87 μm, and CFT of the FB (–) group was 548.08 ± 169.83 μm (*P* < 0.001). SRDH of the FB (+) group was 94.54 ± 127.82 μm, and SRDH of the FB (–) group was 161.28 ± 186.71 μm (*P* = 0.034).

**Table 2:** Pre- and post-treatment best-corrected visual acuity and optical coherence tomographic parameters.

	**FB (+) (*n* = 65)**	**FB (–) (*n* = 100)**	***P***
Mean baseline BCVA (SD), logMAR	0.38 (0.19)	0.59 (0.18)	<0.001^*^
Mean baseline CFT (SD), μm	452.20 (158.97)	541.87 (171.57)	<0.001^*^
Detection of SRD at baseline, n (%)	25 (36.76)	55 (55.00)	0.038^†^
Mean baseline height of SRD (SD), μm	94.54 (127.82)	161.28 (186.71)	0.034^*^
Mean baseline height of non-SRD (SD), μm	357.66 (133.43)	380.59 (150.44)	0.323^*^
Baseline disruption of ELM, n (%)	24 (36.92)	54 (54.00)	0.032^†^
Mean 6M BCVA (SD), logMAR	0.10 (0.16)	0.37 (0.16)	<0.001^*^
Mean 6M CFT (SD), μm	202.95 (23.26)	202.23 (20.38)	0.589^*^
Mean outer nuclear layer thickness (SD), μm	102.15 (14.94)	106.36 (15.35)	0.084^*^
Mean inner segment length (SD), μm	31.78 (2.85)	31.73 (3.22)	0.937^*^
Mean outer segment length (SD), μm	44.09 (5.62)	32.96 (2.99)	<0.001^*^

*FB, foveal bulge; BCVA, best-corrected visual acuity; CFT, central foveal thickness; SRD, serous retinal detachment; ELM, external limiting membrane; 6M, 6 months after the loading treatment; SD, standard deviation*.

*Statistically analysis:*

* *Unpaired Mann-Whitney Test;*

† *Chi-square Test*.

### Post-Treatment BCVA and Optical Coherence Tomographic Parameters

At 6 months, BCVA was significantly better, and OS length was significantly longer in the FB (+) group than the FB (–) group. CFT, ONL thickness, and IS length at 6 months were not significantly different between the two groups (Table 2). BCVA of the FB (+) group was 0.10 ± 0.16 LogMAR, and BCVA of the FB (–) group was 0.37 ± 0.16 LogMAR (*P* < 0.001). There were 40 eyes with a BCVA ≤ 0.0 LogMAR, and 33 (82.50%) eyes with FB, compared to 7 (17.50%) eyes without FB (*P* < 0.001). At 6 months, the OS length of the FB (+) group was 44.09 ± 5.62 μm, and the OS length of the FB (–) group was 32.96 ± 2.99 μm (*P* < 0.001). At 6 months, FB was present in 25 (31.25%) eyes with SRD at baseline, compared to 40 (47.06%) eyes without SRD at baseline (*P* = 0.038).

### Pre- and Post-Treatment Factors Associated With the Existence of FB

The multivariate regression analysis indicated better baseline BCVA, lower baseline CFT, and younger age as significant predictors of the existence of FB at 6 months (Table 3). Better baseline BCVA has the most important contribution to the existence of FB, with an OR of 0.278 (*P* < 0.001) for the FB (+) group vs. the FB (–) group. Moreover, patients with lower baseline CFT (OR = 0.616, *P* = 0.007) and younger age (OR = 0.655, *P* = 0.020) were also significantly associated with the existence of FB at 6 months. The correlations between the existence of FB and post-treatment factors after the resolution of DME were determined by univariate and multivariate logistic regression analysis. The existence of FB was negatively correlated with the post-treatment BCVA (OR = 0.453, *P* = 0.005) and positively correlated with post-treatment OS length (OR = 11.314, *P* < 0.001) in the multivariate regression analysis (Table 3).

**Table 3:** Univariate and multivariate logistic regression analysis of the effect of pre- and post-treatment factors on the presence of foveal bulge at 6 months.

	**Univariate analysis**		**Multivariate analysis**	
	**OR (95% CI)**	***P***	**OR (95% CI)**	***P***
**Pre-treatment factors**				
Mean age, years; quartiles	0.72 (0.54–0.97)	0.029^*^	0.66 (0.46–0.94)	0.020^*^
Mean baseline BCVA, logMAR; quartiles	0.27 (0.17–0.42)	<0.001^*^	0.28 (0.18–0.44)	<0.001^*^
Mean baseline CFT, μm; quartiles	0.62 (0.46–0.83)	0.001^*^	0.62 (0.43–0.88)	0.007^*^
**Post-treatment factors**				
Mean 6M BCVA, logMAR; quartiles	0.24 (0.16–0.36)	<0.001^*^	0.45 (0.26–0.78)	0.005^*^
Mean OS length, μm; quartiles	18.30 (7.57–44.26)	<0.001^*^	11.31 (4.63–27.64)	<0.001^*^

*OR, odds ratio; CI, confidence interval; BCVA, best-corrected visual acuity; CFT, central foveal thickness; 6M, 6 months after the loading treatment; OS, outer segment*.

* *Statistically significant (P < 0.05)*.

## Discussion

In this study, BCVA of eyes with complete integrity of EZ at the fovea ranged from −0.08 to 0.7 LogMAR (20/100–20/16), and 75.76% of these eyes with complete integrity of EZ still had a BCVA > 0.0 LogMAR (20/20) after DME resolution. It appeared that the presence of an intact EZ at the fovea might not be the only biomarker of satisfactory visual prognosis after the resolution of DME. At 6 months, the FB was present in 39.39% of eyes with resolved DME, and BCVA was significantly better in eyes of the FB (+) group than the FB (–) group (Table 2). Moreover, a significant correlation between the existence of FB and BCVA at 6 months was determined by logistic regression analysis (Table 3). Results of this study suggested that the presence of FB could be another reliable biomarker of better post-treatment BCVA in eyes with complete integrity of EZ after the resolution of DME. Consistently, previous studies have shown that eyes with FB had better BCVA after successful retinal detachment repair or resolution of macular edema (10, 11).

In our study, the incidence of SRD and SRDH was significantly lower at baseline in the FB (+) group than the FB (–) group (Table 2). More eyes without SRD had a FB at 6 months than eyes with SRD at baseline (31.25% vs. 47.06%, *P* = 0.038). However, NSRDH was no significant difference at baseline between the two groups. These findings suggested that the presence of FB after the resolution of DME was significantly correlated with the presence and severity of SRD, instead of the severity of non-SRD. Our previous study on the restoration of FB in eyes with resolved SRD-DME also reported similar findings (12).

Serous retinal detachment mainly involves the disruption of outer BRB, whereas non-SRD is caused by the breakdown of inner BRB (13, 15, 16). Since outer BRB is considered to be composed of ELM and the intercellular junction complex of RPE, SRD may be more associated with the photoreceptor damage in DME (16). Consistently, disruption in the photoreceptor integrity is more frequent in patients with SRD, and OS loss is one of the first and major damages resulting from retinal detachment (9, 20, 21). Moreover, Hasegawa et al. have proposed that photoreceptor OS elongation was critical for the presence of FB and the macular edema could cause the absence of FB by damaging the photoreceptors (10, 11). Thus, eyes with SRD seem to undergo more severe OS damage and shortening, which can affect the presence of FB.

Several studies have revealed a significant correlation between the concentrations of interleukin (IL)-6 in the intraocular fluids and serum of patients with DME and the presence of SRD, indicating a significant role of inflammation in the development of SRD (22, 23). Recent studies have shown that the IL-6 signaling pathway plays a prominent role in the pathogenesis of DME by inducing oxidative damage (24, 25). Moreover, as the major cellular source of oxidative stress, photoreceptors are the earliest and primary cellular victims of increased oxidative stress (26). Taken together, inflammation and oxidative stress in eyes with SRD may result in photoreceptor damage and OS loss, further leading to the absence of FB after the resolution of DME (11).

In the present study, several robust predictive biomarkers for the presence of FB in eyes with resolved DME were identified. Baseline BCVA, baseline CFT, and age were significant predictors of the existence of FB at 6 months (Table 3). Patients with better BCVA, lower CFT, and younger age were more likely to have an FB after the resolution of DME. Firstly, better baseline BCVA represented a higher probability for the presence of FB in eyes with resolved DME (OR = 0.278). This result was consistent with previous studies showing a significant correlation between BCVA and OS length before and after anti-VEGF therapy (27, 28). Eyes with better baseline BCVA might experience better photoreceptors regeneration and the restoration of FB after DME resolution. Secondly, higher baseline CFT was a risk factor for the existence of FB in eyes with resolved DME (OR = 0.616). Previous studies had identified baseline CFT as a predictor of anatomic response to anti-VEGF therapy (29, 30). Besides, a lower CFT indicated a less severe macular edema, which might cause less photoreceptor damage, leading to the presence of FB after IVR (11). Thirdly, older age was another risk factor for the existence of FB in eyes with resolved DME (OR = 0.655). This is reasonable since FB is less likely to be observed with increasing age even in normal eyes (18).

There are several limitations to this study. Firstly, this study is a single-site retrospective analysis. Therefore, our findings need to be further validated by prospective multi-center trials. Secondly, the follow-up time was relatively short, and we were unable to perform long-term comparisons between the two groups. Eyes without the presence of FB may experience the restoration of FB and the recovery of BCVA during longer follow-up. Thus, further studies with longer follow-up are needed. Lastly, manual measurement of the OCT parameters by the two ophthalmologists included a subjective process of segment and location, because of no reliable software for automatically measuring the thickness of different retinal layers. However, it should be noted that the ICC suggested a good agreement between the two ophthalmologists.

## Conclusions

In conclusion, the FB could serve as a valuable, easily obtained, and non-invasive biomarker of better visual prognosis after the resolution of DME. The presence of FB after DME resolution was significantly associated with age, baseline BCVA and CFT, and post-treatment BCVA and OS length.

## Data Availability Statement

The raw data supporting the conclusions of this article will be made available by the authors, without undue reservation.

## Ethics Statement

The studies involving human participants were reviewed and approved by the Institutional Review Board of Guangdong Provincial People's Hospital. Since the study is about the retrospective analysis of outcomes of a standard DME treatment and no individual patient could be identified from the data, formal informed consent was waived.

## Author Contributions

HY, YS, SZ, QW, and YH: conception and design. QW, BL, ZL, XZ, YX, and YF: data collection and collation. QW, YH, YYa, YYe, MY, and ZH: data analysis and interpretation. QW and YH: manuscript writing. HY, YS, and SZ: data interpretation and final review of the manuscript. All authors revised and approved the submitted manuscript.

## Funding

This work was supported by Grant 81870663 from the National Natural Science Foundation of China (HY), Grant KJ012019087 of the Outstanding Young Talent Trainee Program of Guangdong Provincial People's Hospital (HY), Grant KJ012019457 from the GDPH Scientific Research Funds for Leading Medical Talents and Distinguished Young Scholars in Guangdong Province (HY), Grant Y012018145 from the Talent Introduction Fund of Guangdong Provincial People's Hospital (HY), Grant WJ2021M224 from the Medical Scientific Research Foundation of Hubei Provincial Health Commission (YS), and Grant A2021378 from the Medical Scientific Research Foundation of Guangdong Province, China (YH).

## Conflict of Interest

The authors declare that the research was conducted in the absence of any commercial or financial relationships that could be construed as a potential conflict of interest.

## Publisher's Note

All claims expressed in this article are solely those of the authors and do not necessarily represent those of their affiliated organizations, or those of the publisher, the editors and the reviewers. Any product that may be evaluated in this article, or claim that may be made by its manufacturer, is not guaranteed or endorsed by the publisher.
